# Comparison of Premixed Human Insulin 30/70 to Biphasic Aspart 30 in Well-Controlled Patients with Type 2 Diabetes Using Continuous Glucose Monitoring

**DOI:** 10.3390/jcm10091982

**Published:** 2021-05-05

**Authors:** Charalampos Margaritidis, Eleni Karlafti, Evangelia Kotzakioulafi, Konstantinos Kantartzis, Konstantinos Tziomalos, Georgia Kaiafa, Christos Savopoulos, Triantafyllos Didangelos

**Affiliations:** 1 Diabetes Center, 1st Propaedeutic Department of Internal Medicine, Medical School, “AHEPA” Hospital, Aristotle University of Thessaloniki, 54636 Thessaloniki, Greece; babismarg14@yahoo.gr (C.M.); linakarlafti@hotmail.com (E.K.); evelinakotzak@hotmail.com (E.K.); ktziomalos@yahoo.com (K.T.); gdkaiafa@yahoo.gr (G.K.); chrisavopoulos@gmail.com (C.S.); 2 Department of Internal Medicine IV, Division of Endocrinology, Diabetology and Nephrology, University of Tübingen, 72076 Tübingen, Germany; Konstantinos.Kantartzis@med.uni-tuebingen.de; 3 Institute for Diabetes Research and Metabolic Diseases (IDM) of the Helmholtz Centre Munich at the University of Tübingen, 72076 Tübingen, Germany; 4 German Center for Diabetes Research (DZD), 72076 Tübingen, Germany

**Keywords:** diabetes mellitus 2, continuous glucose monitoring, premixed human insulin, premixed insulin analog, glycated albumin, fructosamine

## Abstract

Aim: To compare in terms of glycemic variability two premixed insulins, Premixed Human Insulin 30/70 (PHI) and Biphasic Aspart 30 (BiAsp30), using Continuous Glucose Monitoring (CGM) and to estimate the correlation of Glycated Albumin (GA) and Fructosamine (FA) with CGM data. Patients-Data: A total of 36 well-controlled patients with type 2 Diabetes Mellitus (T2DM) underwent 7-day CGM with PHI and subsequently with BiAsp30. GA and FA were measured at the first and last day of each week of CGM. Results: BiAsp30 was associated with lower Average Blood Glucose (ABG) during the 23:00–03:00 period (PHI: 135.08 ± 28.94 mg/dL, BiAsp30: 117.75 ± 21.24 mg/dL, *p* < 0.001) and the 00:00–06:00 period (PHI: 120.42 ± 23.13 mg/dL, BiAsp30: 111.17 ± 14.74 mg/dL, *p* = 0.008), as well as with more time below range (<70 mg/dL) (TBR) during the 23:00–03:00 period in the week (PHI: 3.65 ± 5.93%, BiAsp30: 11.12 ± 16.07%, *p* = 0.005). PHI was associated with lower ABG before breakfast (PHI: 111.75 ± 23.9 mg/dL, BiAsp30: 128.25 ± 35.9 mg/dL, *p* = 0.013). There were no differences between the two groups in ABG, Time In Range and Time Below Range during the entire 24-h period for 7 days, *p* = 0.502, *p* = 0.534, and *p* = 0.258 respectively, and in TBR for the 00:00–06:00 period *p* = 0.253. Total daily insulin requirements were higher for BiAsp30 (PHI: 47.92 ± 12.18 IU, BiAsp30: 49.58 ± 14.12 IU, *p* = 0.001). GA and FA correlated significantly with ABG (GA: r = 0.512, *p* = 0.011, FA: r = 0.555, *p* = 0.005). Conclusions: In well-controlled patients with T2DM, BiAsp30 is an equally effective alternative to PHI.

## 1. Introduction

Type 2 Diabetes Mellitus (DM) is a progressive disorder. It is well known that at the time of diagnosis, the β-cell function is reduced by 50% and relentlessly deteriorates, regardless of the various antidiabetic regimens [1]. Therefore, treatment for type 2 DM constantly needs to be intensified by adding more medications; eventually for many people with type 2 DM, adding insulin to the therapeutic scheme is necessary [2]. The 4-T study demonstrated that a single injection of basal insulin is as effective as a prandial and a premixed insulin in reducing HbA1c, but with less hypoglycemia and weight gain [3]. Therefore, a single injection of basal insulin is the preferred regimen for initiating insulin therapy in type 2 DM [2].

Due to the further deterioration of the β-cell function, many people with type 2 DM on basal insulin need further intensification [3]. At this point postprandial hyperglycemia is the major contributor to patients’ elevated HbA1c, since Fasting Blood Glucose (FBG) is usually well controlled by the basal insulin [4]. Insulin intensification targets postprandial hyperglycemia and can be accomplished either by adding one (basal-plus) or more (basal-bolus) prandial insulin injections, or by adding a GLP-1 receptor agonist (GLP-1 RA) if the patient is not already under such therapy. Alternatively, the basal insulin and all other antidiabetic medications except metformin can be discontinued and a premixed insulin, either human (Premixed Human Insulin-PIH) or analog (Premixed Analog Insulin- PIA) can be initiated [2].

Basal-plus and basal-bolus regimens allow for greater flexibility in the timing of the injections and of the subsequent meals; these regimens closely resemble physiological insulin secretion as well. Adding a GLP1-RA is associated with less hypoglycemia and less weight gain (in some cases even weight loss) [4]. Nevertheless, the use of premixed insulins is still popular in many countries, due to the need for less daily injections and the lower cost. The “INSulin TItration–GAining An understanding of Type 2 diabetes burden in Europe” (INSTIGATE) study demonstrated that premixed insulins are widely used in the UK and Greece, even at insulin naive patients [5], and remain the most popular insulin intensification regimen in Asia [6] and various European countries [7].

Premixed insulins are associated with greater HbA1c reductions than basal insulin alone without increasing overall hypoglycemia [8,9,10,11] or body weight [10,12]; nevertheless, they do increase mild hypoglycemia [8,9,10]. In addition, premixed insulins with two or three daily injections achieve similar HbA1c reductions as basal-plus regimens [13,14,15]. The PREFER study, and a similar study with Continuous Glucose Monitoring (CGM) demonstrated that premixed insulins administered twice daily achieved similar HbA1c reductions as basal-bolus regimens in insulin naive patients, but not in patients already taking insulin [16,17]. On the other hand, participants of the A_1_chieve study who were on basal-bolus regimens and switched to Biphasic insulin Aspart 30/70 (BiAsp30), achieved better metabolic control with less overall, major and nocturnal hypoglycemia. This was attributed by the authors to the better compliance of the patients to the simpler therapeutic regimen [18].

HbA1c is the key determinant used to evaluate the metabolic status of people with DM and the need for further intensification of the therapeutic regimen, because it is closely associated with the future occurrence of complications. Nevertheless, it reflects merely the mean glucose levels of the last three months, which is a relatively long period of time. Fructosamine (FA) and Glycated Albumin (GA) have a higher turnover than HbA1c and reflect the mean glucose levels during the last 3–4 weeks. They are used in situations like gestational and neonatal DM, where there is a need for more frequent monitoring and intensification of the therapeutic regimen [19]. However, all these markers reflect the mean glycemic load of relatively long periods of time, and provide no information for the day-to-day and within-a-day variability of glucose levels as well as for the hyper- and hypoglycemic peaks that can occur, especially in an insulin-treated patient with DM. A normal value of these markers could result either from a constantly well-controlled patient with DM, or from one experiencing alternating periods of hyper- and hypoglycemia. Time in Range (TIR) has been shown to correlate with the risk of microvascular complications [20,21]. Glycemic variability has been related to a greater activation of oxidative stress than sustained euglycemia and has been considered a key factor in the development of endothelial dysfunction and the subsequent changes in microvessels [20]. Continuous Glucose Monitoring (CGM) with the help of glucose sensors can nowadays provide all the necessary clinical information. Currently there are two types of sensors: Professional or reverse type and personal or real-time sensors. Professional sensors are applied for short periods of time for diagnostic purposes. The results are hidden from the patients, and are downloaded by the physicians with the help of special software after the application period. Real time sensors are used for long periods of time, usually together with insulin pumps. Their results are immediately known to the people with DM and help them modify the timing and dosage of insulin accordingly [2,22]. 

The aim of the present study was to compare two premixed insulins, BiAsp30 and PHI 30/70, in terms of glycemic variability (Average Blood Glucose, GA, FA, and Coefficient Variation from CGM), using a 7-day CGM, in patients with type 2 DM in an outpatient setting.

## 2. Materials and Methods

### 2.1. Patients

The study was conducted at the Outpatient Clinic of the Diabetes Centre of the 1st Department of Internal Medicine of “AHEPA” University Hospital in Thessaloniki Greece, from January 2016 until February 2020. Among 120 consecutive type 2 DM patients attending our outpatient clinic from 2016 until the end of 2019, 36 patients were selected according to the following inclusion criteria: (i) Regular attendance at the diabetes clinic every 3 months, (ii) a baseline HbA1c <7%, and (iii) treatment with premixed human insulin 30/70 twice daily with or without metformin. 

Exclusion criteria were: The presence of type 1 DM; stage 4 Chronic Kidney Disease; use of antidiabetic medications other than insulin and metformin; major cardiovascular event (stroke, myocardial infarction, acute coronary syndrome, and peripheral vascular disease) during the last six months; acute illness and hospitalization during the last two weeks; pregnancy; absence of good metabolic control assessed with self-monitoring of blood glucose (SMBG) during the week before entering the study), and the inability of the patients to understand the study procedures and sign the consent form. Good metabolic control is defined as having at least 80% of the minimum 6 daily measurements falling in the range between 80–130 mg/dL preprandially and up to 180 mg/dL postprandially with the Glomerular Filtration Rate estimated using the Chronic Kidney Disease Epidemiology Collaboration (CKD-EPI) equation. The design of this study was in accordance with the Declaration of Helsinki and its latest amendments and all study participants provided informed written consent prior to study enrollment. The Ethics Committee of the School of Medicine, Aristotle University of Thessaloniki approved the study protocol (no. 2279 26.03.2016). The trial was registered at clinicaltrials.gov (identification number NCT04726657). 

### 2.2. Study Design

Upon the initial visit, a full medical history, physical examination, and standard laboratory tests were obtained. This was an open-label study. The patients initially underwent a 7-day CGM (PHI week). Data from the CGM were analyzed and then the patients were switched to BiAsp30 twice daily at a 1:1 insulin unit ratio. BiAsp30 had to be administered 10–15 min before breakfast and dinner, which is 20 min closer to mealtime compared to PHI which is injected 30 min before meals. After titration of the BiAsp30 so that 80% of the minimum 6 daily measurements of SMBG over a week were between 80 and 130 mg/dL, CGM was performed again (BiAsp30 week), and the CGM data were analyzed again, confirming the presence of readings for 7 days. The participants were advised not to change the dose of insulin during the CGM periods, unless a symptomatic hypoglycemic episode had occurred. At the beginning and the end of each week, Glycated Albumin (GA) and Fructosamine (FA) were measured. The primary endpoint of the study was the difference in the Average Blood Glucose (ABG) obtained by the CGM between the PHI and the BiAsp30 week. Secondary endpoints were (i) the difference in the ABG during the night (from 00:00 until 06:00 h) between the PHI and BiAsp30 week, (ii) the difference in the ABG from 23:00 until 03:00 h between the PHI and BiAsp30 week, (iii) the difference in the total units of insulin, and (iv) to determine the most accurate biochemical marker of ABG among FA and GA using all the CGM data collected over the two weeks.

### 2.3. Measurements 

#### 2.3.1. Continuous Glucose Monitoring (CGM)

The 7-day CGM data were obtained using the Medtronic iPRO2 CGM system (Medtronic Diabetes, Northridge, CA, USA). This is a reverse type (professional) sensor, blind to patient and healthcare practitioner at real time, is placed on the abdominal wall or the deltoid area, and measures interstitial glucose every 5 min (288 measurements daily), using a Glucose Oxidase method, for seven days. The iPRO2 can detect glucose concentrations between 40 and 400 mg and needs to be calibrated with data obtained from SMBG. In the first day, patients were asked to measure their Blood Glucose (BG) three times: 1 h after placement of the device, 3 h after placement, and late at night before 00:00. In the subsequent days they were asked to perform SMBG six times: Before and after each of the three main meals and one late at night before 00:00. SMBG was performed using the One Touch Ultra Easy blood glucose monitor (Johnson & Johnson, New Brunswick, NJ, USA). Neither patients nor physicians had access to the CGM data during each week. At the end of each week, data from the iPRO2 were transferred to a PC and analyzed by special software. CGM data were extracted as an Excel file and in an Ambulatory Glucose Profile (AGP) format. The data from the memory of the Glucose Monitor were used to calibrate the CGM system.

#### 2.3.2. Glycated Hemoglobin (HbA1c)

HbA1c was measured at baseline first day of the first week, by routine HPLC immunoassay.

#### 2.3.3. Fructosamine (FA)

Fructosamine was measured using an enzymatic method (Diazyme enzymatic assay—Diazyme laboratories, Poway, CA, USA): Initially serum glycated proteins are broken down to smaller protein fragments with proteinase K, which react with a special fructosaminase, leading to H_2_O_2_ formation. The latter is measured using a photometric assay: Absorbance at 530 nm is proportional to the FA concentration.

#### 2.3.4. Glycated Albumin (GA)

Glycated Albumin was measured using a non-radio labeled ELISA assay (DRG International Inc., Springfield, NJ, USA). GA initially binds with a monoclonal antibody, which specially recognizes the glycated parts of the albumin molecule. After incubation, an enzyme-conjugated polyclonal antibody directed against human albumin is added and the intensity of the color is read in an enzyme-linked immunosorbent assay reader at 450 nm [23]. GA was expressed as a percentage of the total albumin concentration.

#### 2.3.5. CGM Parameters

For each of the PHI and BiAsp30 week, the following values were measured: ABG (mg/dL), Standard Deviation (SD) of ABG (mg/dL), Mean Absolute Relative Difference (MARD) (%), number of high sensor readings (>180 mg/dL), number of low readings (<70 mg/dL), and Area Under the Curve (AUC) above and below range (mg/dL/day). Estimated Glycated Hemoglobin A1c (eA1c) was calculated from the iPRO2 software, and the Coefficient of Variation (CV) was derived from ABG and SD using the (SD/ABG)*100% formula. MARD, which is the average discrepancy between CGM glucose values and simultaneous capillary BG values or venous serum glucose, represents the accuracy of the CGM device. The 24-h period was split into three periods: The day period was between 07:00 and 23:00; the night period (between 23:00 and 07:00) was further divided into two parts, one from 23:00 until 03:00 and one from 03:00 until 07:00. For each of these parts the following parameters were measured: ABG (mg/dL), SD (mg/dL), Time In Range (TIR), Time Above Range (TAR), and Time Below Range (TBR). TIR, TAR, and TBR were expressed in minutes and as a percent of the total CGM time. These parameters were measured for the 00:00–06:00 period as well, since this period of time correlates better with the sleeping pattern of Greek people. For each of the three main meals (breakfast, lunch, and dinner) and for each of the PHI and BiAsp30 weeks, the following parameters were measured: ABG (mg/dL), SD (mg/dL), and time above 180 mg/dL (%) for the 3-h period after each meal.

### 2.4. Statistical Analysis

Statistical analysis was performed using the Statistical Package for Social Sciences (SPSS) version 17.0 for Windows (SPSS Inc., Chicago, IL, USA). The Shapiro–Wilk test was used for normality check. All continuous variables were normally distributed and expressed as mean ± standard deviation. Paired samples t-test was used for comparisons of the CGM data between the two insulin treatment periods. Associations between glycated albumin, fructosamine concentrations at the end of the two interventions, and Average Blood Glucose data from CGM were analyzed with Pearson’s coefficient. *p* < 0.05 was considered significant.

## 3. Results

### 3.1. Baseline Characteristics

The baseline characteristics of the patients enrolled are presented in Table 1. None of the participants had coronary heart disease or retinopathy; 32 were hypertensive (26 patients were receiving angiotensin II receptor blockers, 4 were receiving angiotensin converting enzyme-inhibitors, 24 were receiving calcium channel blockers, 27 were receiving diuretics, and 13 were receiving beta-blockers).

### 3.2. CGM Parameters

The patients during the PHI week received on average a morning insulin dose of 29.0 ± 6.6 IU and an evening dose of 20.6 ± 5.9 IU. After the week in which CGM was performed, each patient was switched to BiAsp30 strictly on a 1:1 insulin dose base. During the following weeks, the BiAsp30 dose was titrated, so that a “good metabolic control” was achieved. This resulted in entering the BiAsp30-CGM week with an average morning dose of 29.8 ± 7.2 IU and an evening dose of 21.4 ± 6.4 IU.

Notably, there were no hypoglycemic events reported by the patients in either of the two weeks.

### 3.3. Main Endpoint

The average blood glucose did not differ between the two insulin regimens (PHI 140.08 ± 20.71 versus BiAsp30 138.08 ± 17.93 mg/dL, *p* = 0.502, Table 2).

### 3.4. Secondary Endpoints 

Average blood glucose during the night (00:00 h–06:00 h) was significantly reduced in the BiAsp30 week compared to the PHI week (111.17 ± 14.74 versus 120.42 ± 23.13 mg/dL, *p* = 0.008) whereas total daily insulin requirements were higher (49.58 ± 14.12 versus 47.92 ± 12.18 IU, *p* = 0.001) (Table 2).

### 3.5. Biochemical Markers of Glycemic Variability

GA and FA concentration on the first and last day of each of the PHI and BiAsp30 week are shown in Table 3.

Average blood glucose was significantly associated with both glycated albumin and fructosamine levels measured at the end of the two insulin regimen weeks (r = 0.512, *p* = 0.011; r = 0.555, *p* = 0.005; respectively). Notably, estimated A1c was significantly associated with both glycated albumin and fructosamine levels (r = 0.558, *p* < 0.001; r = 0.508, *p* < 0.001, respectively).

## 4. Discussion

Our study demonstrates that both insulin regimens (BiAsp30 and PHI based) are equally effective in maintaining good glycemic control in people with type 2 DM, estimated by both CGM and biochemical markers such as FA and GA. 

There were no differences in the accuracy of the two CGM readings as indicated by the MARD values. Furthermore, these values were both very close to 10%; no confirmatory SMBGs are needed below this threshold [24].

In terms of efficacy, BiAsp30 achieved similar ABG and eA1c as PHI. This was expected because of the careful titration of the BiAsp30 before the measurement week. This finding is in concordance with the current literature, regarding both insulin-naive and insulin-treated patients [25,26,27,28,29,30,31], as well as people with type 1 [27,28] and type 2 DM [28,30,32]. On the other hand, Clements et al. in a study of 748 people with type 1 DM and type 2 DM, for 16 weeks found that BiAsp30 was associated with lower HbA1c than PHI. However, in this case, there was a difference in the number of daily injections between the two groups (three for the BiAsp30 group and two for the PHI); there was also an increased number of hypoglycemic events in the BiAsp30 group [31]. Two other studies, which compared BiAsp30 and PHI30/70 with the help of CGM, found no differences in the overall glycemic control between the two groups as our study did [33,34].

In our study PHI was associated with lower ABG before breakfast than BiAsp30. Lower ABG levels before breakfast with PHI have also been found in other studies, but the difference was not statistically significant [28,29,30]. PHI has been reported to be associated with more hypoglycemic events in the morning than BiAsp30 [27]. These findings suggest that PHI is more potent in controlling BG levels several hours after the injection than BiAsp30. There were no differences in ABG three hours after breakfast and before and three hours after lunch and dinner, and in TAR after each meal between BiAsp30 and PIH; this finding is in contrast with various studies demonstrating improved postprandial glycemic control with PIAs [23,27]. In the study by Mortensen et al., BiAsp30 was associated with a lower postprandial increase of blood glucose compared to PHI [27]. Nevertheless, it should be noted that the insulin regimens used in this study were not the same as the ones used in most other studies and in everyday clinical practice: BiAsp30 was used three times daily, before each meal and an additional injection of NPH as basal insulin was given at night; PHI was administered only in the morning, the other meals being covered with regular insulin and an NPH injection was given at night [27]. In the study by Boehm et al., BiAsp30 was associated with lower postprandial glucose levels after all three main meals compared to PHI [28]. Abrahamian et al. found lower BG levels with BiAsp30 before and after dinner and after lunch as well as lower postprandial increase at lunch and breakfast, compared with PHI [30]. Nevertheless, the differences observed in the latter two studies did not lead to a difference in HbA1c, despite the fact that the duration of both studies was long enough for such a difference to occur [28,30]. This leads to the assumption that the clinical significance of the differences in pre- and postprandial BG levels is small. Schmoelzer et al. studied the effects of PIA and PIH after a standard meal in 12 people with type 2 DM and found that PIA was associated with a reduced postprandial peak of BG [35]. Hermansen et al. also studied postprandial BG levels after a standard meal. In this study there was a lispro25/75 group as well. BG levels were measured 5 h after the standard meal and Biasp30 was associated with lower BG levels than both PHI and lispro25/75 [25]. In the study by Ohta et al., BiAsp30 was associated with lower BG levels after breakfast and dinner, and a lower SD of glucose after lunch [34]. It should nevertheless be taken into account that the population of the aforementioned studies had fairly poor glycemic control (HbA1c > 7.8%) [25,27,29,35], meaning that the BG levels could vary widely. In contrast, in our study, all participants had good glycemic control (HbA1c < 7.0%) and, as shown from the CV values for both BiAsp30 and PHI, were found to have relatively stable BG levels. Therefore, the fluctuations of BG levels may have been too narrow for a difference in postprandial BG levels between the two groups to occur. This hypothesis is strengthened by the fact that the study of McNally et al., the population of which had better glycemic control (mean HbA1c 7.5%), compared to the aforementioned studies revealed no differences between BiAsp30 and PHI 30/70 as well [33]. It should also be noted that we did not find a difference in the postprandial glucose levels after breakfast between the two groups, despite the fact that BiAsp30 was associated with higher preprandial BG levels, which indicates that BiaAsp30 may be more potent in controlling postprandial BG levels, at least after breakfast.

We found that BiAsp30 was associated with lower ABG than PHI for the 23:00–03:00 period. Despite the longer TBR in the BiAsp30 time period, no symptomatic hypoglycemic events were reported. TAR for this time period was lower in the BiAsp30 week. On the other hand, for the 03:00–07:00 period, ABG was lower in the PHI week, but the difference was not statistically significant. BiAsp30 was also associated with lower ABG for the 00:00–06:00, presumably due to its stronger action during the first hours of this period. BiAsp30 seems to be more effective during the first four hours of the night, whereas PHI predominates afterwards, the difference being most prominent just before breakfast. To our best knowledge, there are no studies in the current literature with a similar finding. That could be possibly attributed to the special eating habits of Greek people. Greeks tend to eat dinner late, usually after 9:00 p.m., so that the peak effect of the rapid portion of the premixed insulin could match better with the 23:00–03:00 period. In any case, further studies are needed to confirm this finding.

The daily insulin requirements with BiAsp30 were found to be higher than PHI in the present study, but the absolute difference was relatively small; this finding agrees with the results of a subgroup analysis of the IMPROVE study, which also found a small but statistically significant increase in insulin units for patients who switched from PHI to BiAsp30 [36]. McNally et al., also found an increase in the amount of insulin in the BiAsp30 group compared to PHI, but the difference was not statistically significant [33]. On the other hand, in a comparative study by Balaji et al., in 76 women with GDM, no differences in the units of insulin between BiAsp30 and PHI 30/70 were reported [37]. 

In our study, BG levels were generally within the range of 70–180 mg/dL during both PIA and PHI weeks. TIR was over 70% for both groups not only for the entire 24 h period, but also during the night and there were no differences between BiAsp30 and PHI. TIR over 70% is another marker of good glycemic control and the mean TIR values in our study lie within the target proposed by the American Diabetes Association in its current guidelines [2]. To the best of our knowledge, the present study is unique in the literature because the good glycemic control was by design an inclusion criterion. In addition, since the insulin analog BiAsp30 was not used for improving diabetes control, our study allows for a better understanding of the pharmacokinetics of the premixed insulin preparations.

The number of events outside the BG range of 70–180 mg/dL was generally small. Nevertheless, there was a trend towards a longer duration of hypoglycemic events on average during the BiAsp30 week as indicated by the time spent in hypoglycemic events and AUC < 70 mg/dL. Therefore, we do not consider this trend to be necessarily significant from a clinical perspective because not a single symptomatic hypoglycemic event was reported by any of the patients. There were no differences between BiAsp30 and PHI. The majority of these events were in the hyperglycemic range; hypoglycemic events were rare and hypoglycemic events < 54 mg/dL even fewer, with no differences between the two groups as well.

Furthermore, there were no differences in TAR and AUC for glucose concentrations > 180 mg/dL between the two groups for the entire 24-h period, the 00:00–06:00 period and the 3-h period after each meal as well. It is noteworthy that even our participants, who had good glycemic control with narrow fluctuations, spent about 20% of their time in the hyperglycemic range (BG > 180 mg) regardless of their insulin regimen. This finding agrees with Li et al. who demonstrated that patients with long standing DM spent 16–18% of their time in the hyperglycemic region. Patients with newly diagnosed DM spent 12–14% in hyperglycemia and the difference compared to long-standing DM was statistically significant [17]. The progressive decline of the β-cell function and consequently of endogenous insulin production in people with long-standing DM could possibly explain this finding.

Time spent in hypoglycemia, expressed as TBR, was not different between the two groups as well. That accounts for the entire 24 h period, as well as for each of the two out of three-night periods studied (03:00–07:00 and 00:00–06:00); for the 23:00–03:00 period, TBR was significantly higher with BiAsp30 than with PHI. There were also no differences in time spent in grade-2 hypoglycemia (<54 mg/dL), or in the total number of hypoglycemic events. This finding agrees with a meta-analysis of 45 studies with 14.603 participants, which found no differences in mild and serious hypoglycemic events between PHI and PIA [38]. In respect of other studies that used CGM, Ohta et al. reported no events of BG < 70 mg/dL [34], probably due to the fact that the participants of the study had rather poor glycemic control (mean HbA1c 8.4%). McNally et al. found no differences in hypoglycemia < 54 mg/dL, total hypoglycemic events, and events during the daytime period, but BiAsp30 was associated with less hypoglycemic events and less time spent in mild hypoglycemia during the night compared to PHI; nevertheless, the absolute difference between the two groups was rather small (mean number of nocturnal events 1.18 vs. 1.56 for BiAsp30 and PHI respectively). It is interesting that the BiAsp30-related events occurred earlier than PHI-related events (peak number of events at 03:00 for BiAsp30 vs. 06:00 for PHI) [33]. This agrees with our finding of a higher TBR with BiAsp30 during the 23:00–03:00 period, which, taken together with the lower ABG with BiAsp30 during the same period, strengthens the assumption that BiAsp30 has stronger action than PHI during the first part of the night. Furthermore, the overall number of such events for both groups was small and an observation for longer periods of time is required for such differences, if they exist, to occur. Boehm et al., demonstrated that PIAs were associated with less serious hypoglycemic events than PHI after the second year of treatment [28], a time frame far beyond any existing in the literature of CGM study.

Our study demonstrated that there are no important ‘quantitative’ differences between BiAsp30 and PHI regarding overall glycemic control. However, evidence from other studies suggest that there are ‘qualitative’ differences in favor of PIAs. As a result of their faster onset of action, no lag period before the start of the meal is needed. They are administered immediately before and in special cases after a meal, which leads to increased patient satisfaction and compliance to treatment. A post-hoc analysis of the A_1_chieve study [18] demonstrated that people who had switched to BiAsp30 from PHI had improved Quality of Life (QoL), measured by the visual analog scale score, after 24 weeks [39]. The transition from PHI to BiAsp30 led to improved glycemic control in the A_1_chieve study [18], which may explain the increase in patient satisfaction. Even, in the study of Balaji et al., where there were no differences in the glycemic control, patients were more satisfied with BiAsp30, because of its more convenient administration before meals [37,38]. In a study by Yamada et al., PIA (in this case lispro50/50) led to improved glycemic control compared with PHI, but there were no differences in QoL between the two groups [40], thus strengthening the hypothesis that there are other factors beyond glycemic control contributing to overall patient satisfaction and QoL. In our study we also had generally positive feedback from the patients regarding the use of BiAsp30, mainly because of the time of injection however we did not use specific tools to formally report this feedback and therefore we are not able to perform an appropriate QoL analysis.

Finally, we found no differences in the biochemical markers of GV (GA and FA) between the two groups. This was expected since there were very small differences in the CGM data between the two groups.

Both biochemical markers of GV (GA and FA) correlated significantly with ABG derived from CGM. To our best knowledge, there are few data on this point in the current literature and most of them come from special populations with type 2 DM. Beck et al., in a study using CGM in 26 children with type 1 DM and mean baseline HbA1c 7.5%, found a similar correlation for HbA1c, GA and FA, the strongest with HbA1c [41]. On the other hand, Chan et al., in a similar study in 56 children with pre-diabetes and type 2 DM with mean HbA1c 5.9% found that FA had the strongest correlation with ABG, followed by HbA1c and GA [42]. Desouza et al. used 4 weeks of CGM data from 34 people with DM (10 type 1 and 21 type 2) and mean HbA1c 9.5% and found that GA correlated with the CGM data stronger than ABG [43]. Divani et al., in a CGM study with 37 people with DM and end-stage Chronic Kidney Disease undergoing dialysis demonstrated that GA exhibited the strongest correlation with ABG from the CGM, when ABG was greater than 184 mg/dL [44]. These studies suggest GA may correlate stronger with ABG in poorly controlled DM, whereas in well-controlled DM, HbA1c and FA may be superior.

One of the strengths of our study is that both BiAsp30 and PHI were tested in the same population and thus in the same conditions regarding glycemic control and the duration of DM and probably the same type of diet. No standard meal was used; both premixed insulins were tested in real-life circumstances, in terms of the number, contents, and the timing of the meals. The duration of the CGM is, to our best knowledge, longer than most other studies using professional glucose sensor. The participants all had good glycemic control and therefore, the findings of our study can be attributed only to the special pharmacodynamic and pharmacokinetic properties of each premixed insulin.

One weakness of our study is the relatively small sample size. Furthermore, our study has no formal cross-over design, and in this sense our findings may be considered with less evidential competence. However, we have data from 7 consecutive days for each patient, which provided us with an abundance of measurements, while older studies took in account data of only 3 days (older CGM-device). We feel that this enhances the reliability of our findings. Due to the study design, hyper- and hypoglycemic events in both groups were rare. Therefore, although the observation period was longer than many other similar studies, an even longer observation period may be needed for differences between the two groups to occur.

## 5. Conclusions

In well-controlled people with type 2 DM, BiAsp30 has similar efficacy and GV compared to PHI, without differences in the number and duration of hyper- and hypoglycemic events. BiAsp30 achieved lower ABG, less TAR, and more TBR in the 23:00–03:00 period, and PHI achieved lower ABG levels before breakfast. Hypoglycemia < 54 mg/dL was rare and there were no differences between the two groups. Given the fact that BiAsp30 is more convenient than PHI, which leads to increased compliance, BiAsp30 evolves as a more attractive option for the treatment of people with DM, especially in elderly people with various comorbidities that limit their ability to follow complex therapeutic regimens.

## Figures and Tables

**Table 1:** Patients’ baseline characteristics.

Patients’ Characteristics	
**N**	**36**
Gender (men/women)	18/18
Age (years)	61.1 ± 8.5
Duration of Diabetes Mellitus (years)	10.6 ± 6.2
Βody Μass Ιndex (Kg/m^2^)	29.0 ± 3.3
HbA_1_c (%)	6.69 ± 0.16
Serum albumin (g/L)	4.64 ± 0.47
eGFR (mL/min) *	81.08 ± 16.96

* eGFR was calculated with the Chronic Kidney Disease Epidemiology Collaboration (CKD-EPI) equation. Abbreviations: HbA1c: Glycated Hemoglobin A1c.

**Table 2:** A summary of the key CGM parameters for each of the PHI and BiAsp30 weeks.

CGM Parameters	PHI Week	BiAsp30 Week	*p*
ABG (mg/dL)	140.08 ± 20.71	138.08 ± 17.93	0.502
Estimated HbA1c (eA1c) (%)	6.51 ± 0.72%	6.44 ± 0.61%	0.474
SD (mg/dL)	45.92 ± 12.56	48.17 ± 19.16	0.452
CV (%)	32.49 ± 6.71	34.18 ± 11.44	0.274
MARD (%)	12.558 ± 5.22	11.067 ± 6.32	0.090
**Number of events outside the range of 70–180 mg/dL**			
Total events	17.83 ± 7.34	19.08 ± 6.30	0.130
Hyperglycemic events (>180 mg/dL)	13.67 ± 6.42	13.58 ± 6.31	0.915
Hypoglycemic events (<70 mg/dL)	4.17 ± 3.55	5.5 ± 3.40	0.063
Hypoglycemic events (<54 mg/dL)	1.08 ± 1.13	1.83 ± 3.30	0.134
**Time spent in and outside the range of 70–180 mg/dL**			
TIR (% of the total CGM time)	75.92 ± 12.42	74.17 ± 15.90	0.534
TAR (% of the total CGM time)	19.42 ± 13.85	19.58 ± 10.75	0.940
AUC for glucose concentrations > 180 mg/dL	8.11 ± 7.55	9.93 ± 9.62	0.385
TBR (% of the total CGM time)	4.68 ± 5.33	6.25 ± 8.88	0.258
AUC for glucose concentrations < 70 mg/dL	0.65 ± 0.79	6.15 ± 17.77	0.070
Time spent in hypoglycemia < 54 mg/dL (min)	17.88 ± 33.92	28.88 ± 80.45	0.529
AUC for glucose concentrations < 54 mg/dL	0.11 ± 0.26	0.21 ± 0.63	0.410
**Post-meal glycemic variability (3-h period)**			
**Breakfast**			
ABG preprandial (mg/dL)	111.75 ± 23.9	128.25 ± 35.9	**0.013**
ABG 180 min (mg/dL)	169.92 ± 40.80	178.17 ± 43.22	0.369
ΔABG (mg/dL)	58.17 ± 36.91	49.92 ± 38.55	0.207
SD 0 min (mg/dL)	23.83 ± 13.14	25.92 ± 22.4	0.411
SD 180 min (mg/dL)	43.17 ± 14.19	44.08 ± 13.55	0.699
TAR (%)	40.33 ± 31.23	44.67 ± 26.24	0.458
**Lunch**			
ABG preprandial (mg/dL)	139.33 ± 28.11	133.75 ± 21.06	0.348
ABG 180 min (mg/dL)	151.33 ± 26.05	153.67 ± 24.83	0.539
ΔABG (mg/dL)	12.00 ± 29.93	19.92 ± 33.24	0.305
SD 0 min (mg/dL)	32.83 ± 15.12	27.83 ± 18.83	0.141
SD 180 min (mg/dL)	38.50 ± 16.39	35.08 ± 13.68	0.336
TAR (%)	26.83 ± 25.11	27.00 ± 17.11	0.970
**Dinner**			
ABG preprandial (mg/dL)	149.42 ± 30.94	151.58 ± 28.20	0.717
ABG 180 min (mg/dL)	146.58 ± 29.80	143.75 ± 33.51	0.493
ΔABG (mg/dL)	-2.83 ± 25.49	-7.83 ± 20.59	0.391
SD 0 min (mg/dL)	39.33 ± 17.29	32.42 ± 31.00	0.210
SD 180 min (mg/dL)	34.67 ± 15.88	33.58 ± 17.63	0.667
TAR (%)	27.09 ± 30.25	23.45 ± 22.18	0.340
**Night period (sum of the week)**			
**23.00–03.00**			
ABG (mg/dL)	135.08 ± 28.94	117.75 ± 21.24	<**0.001**
SD (mg/dL)	37.58 ± 13.27	35.42 ± 13.87	0.164
TIR (70–180 mg/dL) (%)	78.63 ± 17.85	77.41 ± 16.38	0.706
TAR (>180 mg/dL) (%)	17.71 ± 18.37	11.46 ± 7.79	**0.021**
TBR (<70 mg/dL) (%)	3.65 ± 5.93	11.12 ± 16.07	**0.005**
**03.00–07.00**			
ABG (mg/dL)	102.17 ± 34.81	108.25 ± 19.07	0.441
SD (mg/dL)	30.67 ± 14.42	28.83 ± 25.43	0.592
TIR (70–180 mg/dL) (%)	80.59 ± 15.61	83.71 ± 23.14	0.493
TAR (>180 mg/dL) (%)	5.39 ± 9.80	4.03 ± 8.40	0.505
TBR (<70 mg/dL) (%)	14.01 ± 15.10	12.24 ± 13.18	0.674
**00.00–06.00**			
ABG (mg/dL)	120.42 ± 23.13	111.17 ± 14.74	**0.008**
SD (mg/dL)	36.00 ± 14.74	34.17 ± 19.68	0.441
TIR (70–180 mg/dL) (%)	91.54 ± 8.81	87.97 ± 16.13	0.653
TAR (>180 mg/dL) (%)	0	0	--
TBR (<70 mg/dL) (%)	8.45 ± 8.81	12.01 ± 16.13	0.253
Time spent in hypoglycemia (<54 mg/dL) (%)	1.00 ± 2.11	2.84 ± 9.04	0.136

Abbreviations: CGM: Continuous Glucose Monitoring, eHbA1c: Estimated glycated hemoglobin. Key continuous glucose measurements for each of the two premixed insulins. PHI: Premixed Human Insulin, PIA: Premixed Insulin Analog, ABG: Average Blood Glucose, SD: Standard Deviation, CV: Coefficient of Variation, MARD: Mean Absolute Relative Difference, TIR: Time In Range, TAR: Time Above Range, TBR: Time Below Range, AUC: Area Under The Curve, ΔABG: Change of ABG after a meal). Significant results are shown in bold. For an explanation see Section 2.3.5.

**Table 3:** Biochemical indices of glycemic variability on day 1 and day 7 of each week.

	PHI Week	BiAsp30 Week	*p*
**GA (%)**			
Day 1	8.40± 2.13	8.39 ±1.78	0.994
Day 7	8.43± 2.79	8.31± 1.95	0.706
**FA (μmol/L)**			
Day 1	292.58± 53.28	300.33± 57.63	0.151
Day 7	295.83 ± 75.54	293.75 ± 52.50	0.700

Abbreviations: PHI: Premixed Human Insulin, BiAsp30: Biphasic Aspart 30.

## Data Availability

Data are available upon request.
